# Chronic Hepatitis B Virus Infection Acquired Under Cytostatic Treatment in Childhood — Clinical, Virological and Immunological Long‐Term Follow‐Up

**DOI:** 10.1111/jvh.70111

**Published:** 2025-12-06

**Authors:** Thomas Baumgarten, Felix Lehmann, Tina Senff, Heiko Slanina, Christian G. Schüttler, Andreas Walker, Wolfram H. Gerlich, Reinald Repp, Jörg Timm, Dieter Glebe, Markus Metzler

**Affiliations:** ^1^ Department of Paediatrics and Adolescent Medicine University Hospital Erlangen & Friedrich‐Alexander‐Universität Erlangen‐Nürnberg (FAU) Erlangen Germany; ^2^ Department of Paediatric Haematology and Oncology University Children's Hospital Tuebingen Tuebingen Germany; ^3^ Charité‐Universitätsmedizin Berlin, Corporate Member of Freie Universität Berlin and Humboldt‐Universität Zu Berlin Institute of Virology Berlin Germany; ^4^ Institute of Medical Virology Justus Liebig University Giessen Giessen Germany; ^5^ National Reference Centre for Hepatitis B and D Viruses Giessen Germany; ^6^ German Center for Infection Research (DZIF), partner Site Giessen‐Marburg‐Langen Giessen Germany; ^7^ Institute of Pathology and Molecular Pathology, Helios University Clinic Wuppertal Witten/Herdecke University Witten Germany; ^8^ Institute of Virology, Medical Faculty and University Hospital Düsseldorf Heinrich‐Heine‐University Düsseldorf Germany; ^9^ Children's Hospital Fulda Fulda Germany; ^10^ Comprehensive Cancer Center Erlangen‐EMN (CCC ER‐EMN) Erlangen Germany

**Keywords:** chemotherapy, chronic HBV infection, immunosuppression, T‐cell response, viral mutations

## Abstract

Oncology patients receiving cytostatic therapy used to be at high risk of HBV infection when HBV screening measures were less reliable. Infections acquired under these conditions often persist, like those acquired perinatally or during early infancy. We studied the long‐term clinical outcomes, viral characteristics, and virus‐specific T‐cell immunity of chronic HBV infection acquired during chemotherapy. We examined 16 chronically HBV‐infected former paediatric oncology patients who were infected during cytostatic treatment in the 1980s. Patients underwent physical examination, laboratory liver function testing, non‐invasive measurement of liver stiffness, and determination of HBV serology and DNA levels. If the material was sufficient, HBV sub‐genotype, drug resistance and immune escape mutations, and mutations associated with HBeAg negativity were analysed. The frequency of HBV core‐specific CD8+ T cells was measured after in vitro antigen‐specific expansion. All but one patient were chronically infected with detectable HBsAg but were HBeAg‐negative, mostly with low viraemia. Four patients were under ongoing effective antiviral therapy, and four required treatment initiation due to high viraemia or advanced liver disease. Hepatic effects were predominantly observed in highly viraemic patients. No drug resistance or immune escape mutations were observed. In two highly viraemic patients, basal core promoter and precore region mutations reducing HBeAg expression were identified. HBV core‐specific CD8+ T cells were detected in all patients, but their frequency was low. In conclusion, more than 30 years after primary HBV infection was acquired during chemotherapy, the course of infection still resembles that of perinatally acquired infections.

**Abbreviations**
ALT: alanine transaminase
anti‐HBc: hepatitis B core antibody
anti‐HBe: hepatitis B e antibody
anti‐HBs: hepatitis B surface antibody
BCP: basal core promoter
CD: cluster of differentiation
CEF: human cytomegalovirus, Epstein–Barr virus, and influenza A virus (“flu”)
CHB: chronic hepatitis B
EASL: European Association for the Study of the Liver
HBeAg: hepatitis B e antigen
HBsAg: hepatitis B surface antigen
HBV: hepatitis B virus
HBx: hepatitis B virus regulatory protein X
HCV: hepatitis C virus
HDV: hepatitis D virus
HEV: hepatitis E virus
IFN‐γ: interferon γ
LoD: limit of detection
METAVIR: Meta‐analysis of Histological Data in Viral Hepatitis
preC: precore region

With over 300 million people infected worldwide [1], chronic HBV (hepatitis B virus) infection is a persistent global health burden that causes substantial morbidity and mortality through associated complications, particularly liver cirrhosis and hepatocellular carcinoma. Chronic HBV infection is defined as the detectability of HBsAg (hepatitis B surface antigen) for more than six months after infection, indicating failure of immunologic control and clearance of the virus. In contrast to the predominant acute, self‐limiting course of HBV infection in adolescents and adults, chronic HBV infection is mainly observed after neonatal infection or in early infancy: Chronicity rates are about 90% in perinatally infected babies, 25%–50% in children infected at 1–5 years of age, and subsequently decline towards < 5% in adolescents and adults [2, 3, 4, 5].

The natural history of chronic HBV infection, acquired perinatally or in early childhood, is characterised by an initial phase of high replication, low inflammation, and HBeAg (hepatitis B e antigen) positivity, which usually lasts until early adulthood. Loss of HBeAg occurs in approximately half of the cases [6], sometimes accompanied by hepatitis flares. Subsequently, HBV replication declines as a sign of partial viral control. However, sustained, complete infection control with seroconversion of HBsAg to anti‐HBs (hepatitis B surface antibody), the so‐called “functional cure”, is a rare event in chronic HBV infection [7]. As immunologic selective pressure increases and HBV is progressively cleared from the body, HBeAg‐negative viral variants emerge [8]. Various mutations in the BCP (basal core promoter) and preC (precore) regions have been described as the molecular basis for HBeAg negativity. Although HBV shows relatively high sequence heterogeneity due to replication by reverse transcription, with at least nine different genotypes described to date [9], a certain level of intrahost genetic stability over time in chronic infections has been reported [10].

Several immunologic mechanisms facilitating chronic HBV infection have been described [11]; in particular, virus‐specific CD8+ (cluster of differentiation) T lymphocytes play a pivotal role in achieving sustained viral control by cytolytic and non‐cytolytic (cytokine‐mediated) effects. In chronically infected patients, T‐cell dysfunction has been well described, possibly as a consequence of exhaustion or anergy in the context of permanent overstimulation of HBV‐specific T‐cell signalling and upregulation of inhibitory and inactivating pathways [12]. Moreover, the selection of mutations in viral epitopes targeted by CD8+ T cells may further contribute to immune escape [13]. However, the frequency of these cells declines when HBsAg clearance is achieved [14]. Depending on the stage of HBV infection, low frequencies of HBV‐specific CD8+ T cells are common in chronically infected patients.

HBV infection presents a significant risk to all patient groups with dysfunctional immune defences, whether due to disease or iatrogenic causes. Oncology patients were among the first in whom HBV was described [15]. There is extensive knowledge of HBV reactivation during pharmaceutical immunosuppression [16] such as cytostatic or immunomodulatory therapy; however, little systematic research and experience exist regarding the course of primary HBV infection acquired under cytostatic treatment. The cohort examined in this study is exceptional, as it consists of former paediatric oncology patients who have acquired a primary HBV infection during their cytostatic treatment, all of them with an identical virus strain. Preexisting older data from this cohort suggest a course similar to that of perinatally acquired infections [17, 18]. The current work aims to determine whether the long‐term clinical course of this unique group of chronic HBV infections acquired under cytostatic treatment still exhibits the characteristics of a perinatally acquired infection over 30 years later. Furthermore, the emergence of viral mutations under these circumstances and virus‐specific T‐cell immunity was examined.

## 1 Materials and Methods

### 1.1 Patient Population

We included 16 individuals who had been infected with HBV during an outbreak between 1983 and 1986, affecting 74 patients aged 3–18 years who were undergoing cytostatic chemotherapy at the Department of Paediatric Haematology and Oncology in Giessen, Germany [19]. All patients were infected with an identical virus strain (Genotype D1 [20], HBsAg subtype ayw2, EMBL accession number: Y07587 [21]), suggesting a single source of infection and patient‐to‐patient transmission rather than a transfusion‐transmitted infection [22]. Until 1989, 20 children died of their malignancy, with no negative prognostic influence of HBV infection in cases with acute lymphoblastic leukaemia [23]. Five years after termination of cytostatic treatment, > 90% of the surviving patients (49 out of 54) still showed a high replicative, low inflammatory HBeAg‐positive chronic HBV infection (formerly termed “immune tolerant carrier state” [24]), while < 10% (5 out of 54) showed HBsAg to anti‐HBs seroconversion, indicating functional cure. Remarkably, anti‐HBc (hepatitis B core antibody) was not detected in the serum of 20% of the patients during the first follow‐up period and was negative on repetitive testing for several years [18]. None of the patients had elevated liver enzyme levels that exceeded the intermittent, slight elevations typically observed during chemotherapy. Liver biopsies taken from 36 patients in 1989 only showed signs of “minimal hepatitis” in some cases [18].

**FIGURE 1:**
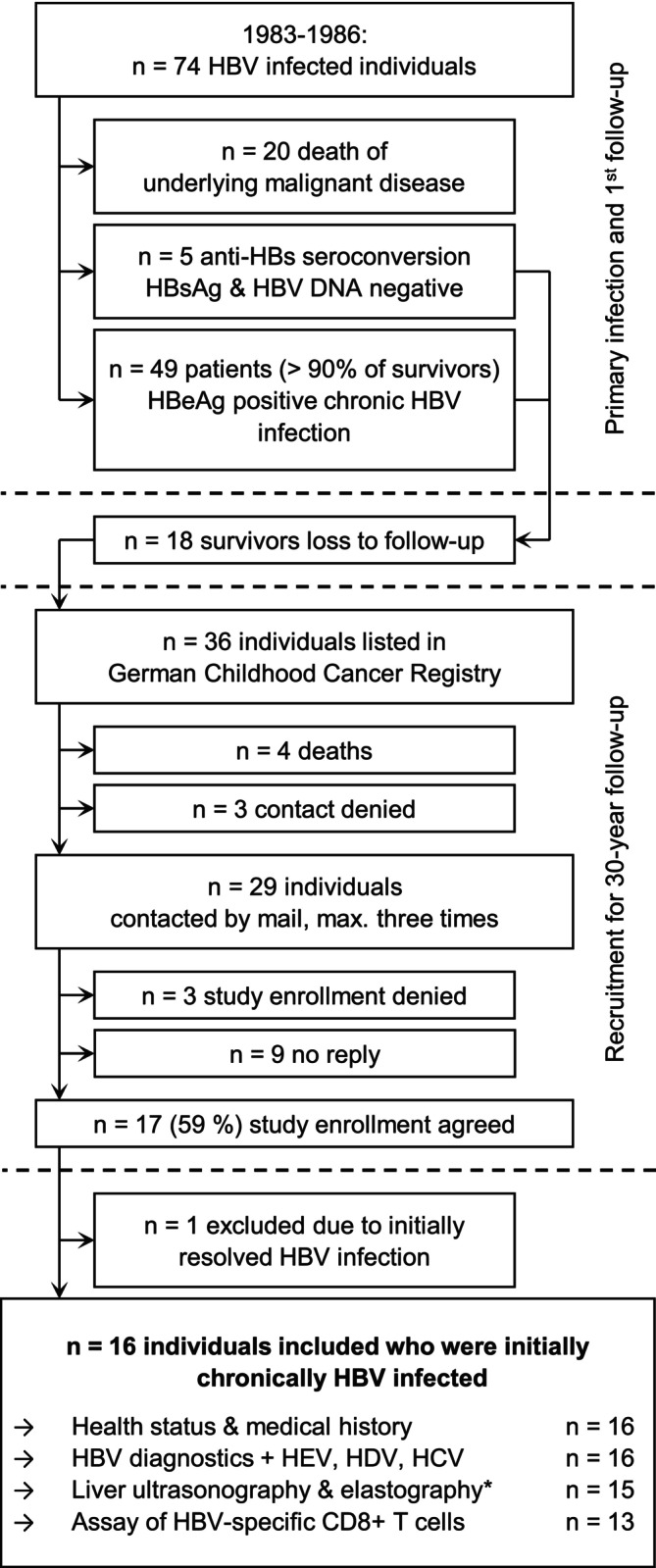
Cohort diagram. *Liver elastography was performed using Acoustic Radiation Force Impulse (ARFI) imaging technology. Anti‐HBs, hepatitis B surface antibody; HBeAg, hepatitis B e antigen; HBsAg, hepatitis B surface antigen; HBV, hepatitis B virus; HCV, hepatitis C virus; HDV, hepatitis D virus; HEV, hepatitis E virus.

Figure 1 provides an overview of the cohort and recruitment process. We were able to contact 29 former survivors via the German Childhood Cancer Registry. Seventeen (59%) agreed to participate, of which 16 were included in the study. The 17th person was one of the five survivors who initially had anti‐HBs seroconverted; i.e., they had experienced an acute HBV infection and never developed a chronic infection.

Written consent was obtained from all participants, and all research was conducted in accordance with both the Declarations of Helsinki and Istanbul. The study protocol was approved by the ethical review committee of Friedrich‐Alexander University, Erlangen‐Nuremberg (Reference No: 126_19 B).

### 1.2 Clinical Follow‐Up

All patients were interviewed, and pre‐existing medical findings were collected from the patients and attending physicians. Serum tests were performed in Erlangen and Giessen. Liver enzymes, bilirubin, albumin, plasma coagulation markers (international normalised ratio and activated partial thromboplastin time), electrolytes (Na^+^, K^+^, Ca^2+^, Cl^−^, PO_4_^−^, and Mg^2+^), and HBV markers were tested. In the presence of HBsAg, HBV DNA levels were also determined (see 1.3 *Virologic assessment*). Furthermore, we determined the IgG antibody titres against HCV (hepatitis C virus) and HEV (hepatitis E virus) and total antibody titres (IgG and IgM) against HDV (hepatitis D virus). In the case of positive HEV IgG, IgM antibody and RNA levels were determined to rule out acute infection. Subsequently, 15 patients were medically examined by hepatologists at the departments of Internal Medicine of the University Hospital Erlangen, Germany or University Hospital Giessen, Germany, including abdominal ultrasonography with non‐invasive measurement of liver stiffness using Acoustic Radiation Force Impulse imaging (Acuson S2000 Virtual Touch tissue quantification, Siemens Healthineers). The cut‐off values for the application of the METAVIR (Meta‐analysis of Histological Data in Viral Hepatitis) fibrosis score [25] to elastography results were adopted from Friedrich‐Rust et al. [26] For the findings of abdominal ultrasonography, we defined two categories: “steatosis” and “parenchymal damage” (i.e., signs of fibrosis or cirrhosis).

The phase of chronic HBV infection was determined using serological HBV parameters and ALT (alanine transaminase) levels, and, if available, a non‐invasive assessment of liver disease, following the nomenclature introduced in 2017 by the EASL (European Association for the Study of the Liver) [24]. The indications for antiviral treatment were defined according to the current clinical practice guidelines of the EASL [24].

### 1.3 Virologic Assessment

The qualitative determinations of HBsAg (HBsAg Qualitative II, LoD (Limit of detection) 0.02 IU/mL), HBeAg, anti‐HBc (Anti‐HBc II, LoD 0.5 PEI U/mL), anti‐HBe (hepatitis B e antibody) as well as the quantitative determinations of anti‐HBs (LoD 0.98 IU/L) and HBsAg (LoD 0.05 IU/mL; carried out only if qualitatively tested positive) were performed by commercial chemiluminescent microparticle immunoassay (ARCHITECT System, Abbott). Anti‐HDV total antibodies were tested using a chemiluminescent immunoassay (LIAISON murex Anti‐HDV, DiaSorin), anti‐HCV virus total immunoglobulin with the ARCHITECT System (Abbott), and anti‐HEV IgG and IgM antibodies were determined using ELISA (HEV ELISA 4.0 and HEV IgM ELISA 3.0, MP Biomedicals). HEV RNA was quantitatively assayed using reverse transcription PCR (RealStar HEV RT‐PCR Kit 2.0, Altona). HBV DNA was quantified using real‐time PCR (LoD < 10 IU/mL, 1 IU/mL = 5.4 genomes/mL, linear range 20 to 1.7x10^8^ IU/mL, Cobas TaqMan HBV Test, Roche).

If the DNA concentration was ≥ 35 IU/mL, a 621 bp fragment of the HBV genome spanning the S and polymerase gene regions (s74 to s226 and rt83 to rt288, respectively) was amplified by PCR as previously described [27] for Sanger sequencing (Eurofins Genomics) and subsequent genotyping and mutational analysis. The 621 bp fragment allows screening for immune escape and drug resistance mutations, as it encodes the major hydrophilic region of the S protein, including the *a* determinant, which is believed to be a main target of the antibody‐mediated immune response, and the main part of the reverse transcriptase domain of the DNA polymerase protein, which is the target of nucleos(t)ide analogues used in HBV therapy. Genotyping was performed based on the aforementioned DNA sequence using the Geno2Pheno online tool (https://www.geno2pheno.org/). The polymerase and S gene overlapping open reading frames were analysed for known drug resistance mutations [28] and immune escape mutations [27], respectively. To determine the molecular basis of HBeAg negativity, we sequenced the BCP and preC regions to characterise mutations known to reduce or abolish HBeAg expression. HBV relaxed circular DNA from virions in serum samples was purified using the innuPREP Virus DNA/RNA kit (Analytik Jena) and then amplified by hemi‐nested PCR spanning the BCP, preC, and entire Core open reading frame using a primer set modified from Sato et al. [29] (outer forward primer 5′‐CAT AAG AGA CTC TTG GAC T‐3′, inner forward primer 5′‐TGT CAA CGA CCG ACC‐3′, and reverse primer 5′‐GGA AAR GAD GGD GTT TDC C‐3′) and Platinum Superfi II Polymerase (Thermo Fisher Scientific). The PCR products were subjected to agarose gel electrophoresis and gel extraction (Nucleospin Gel Extraction Kit, Takara Bio), followed by Sanger sequencing (LGC genomics). If no amplificates were obtained from the PCR outlined above, in a second attempt, the reverse primer was exchanged (5′‐AGA CTC TAA GGC TTC CCG‐3′), resulting in shorter amplificates spanning only BCP, preC, and one‐third of the Core open reading frame. In a third attempt, DNA was subjected to nested PCR as described by Sato et al. [29]

### 1.4 HBV‐Specific CD8+ T‐Cell Response

The HBV core‐specific reactivity of cytotoxic T cells was tested in 13 patients; no samples could be obtained from the remaining three patients. IFN‐γ (interferon γ) secretion by CD8+ T cells was measured using flow cytometry after in vitro antigen‐specific expansion and intracellular cytokine staining as previously described [30]. Briefly, peripheral blood mononuclear cells were isolated from EDTA‐anticoagulated blood samples by density gradient centrifugation (ROTISep 1077 human; Carl Roth GmbH, Germany), conserved in 40% fetal bovine serum (FBS), 40% RPMI, and 20% DMSO, and stored at −80°C until further analysis. A total of 21 18mer peptides (EMC, Tübingen, Germany) covering the precore/core region of HBV genotype D were divided into four pools and used for antigen‐specific stimulation of CD8+ T cells. A CEF (human cytomegalovirus, Epstein–Barr virus, and influenza A virus) peptide pool (peptides & elephants, Germany) was used as a positive control. After thawing, the cells were suspended in complete medium R10 (RPMI 1640 containing 10% fetal calf serum, 100 U/mL penicillin, 100 μg/mL streptomycin, and 10 mmol/L HEPES buffer) for stimulation with HBV core peptides (1 μg/mL) in the presence of IL‐2 (25 U/mL, Roche, Germany) and anti‐CD28/CD49d (0.5 μg/mL, BD Biosciences, Germany). Fresh medium and IL‐2 were added after 7 days. After 14 days of antigen‐specific expansion, CD8+ T cells were re‐stimulated with the same peptide pool and tested for IFN‐γ secretion using flow cytometry on a FACSCanto (BD Biosciences, Germany) after intracellular cytokine staining with anti‐human IFN‐γ (BD Biosciences, Germany). The percentage of IFN‐γ+ CD8+ T cells detected in the unstimulated culture (negative control) was subtracted from the results of the stimulated culture to obtain the actual percentage of IFN‐γ+ CD8+ T cells reactive to the CEF peptide pool or the different HBV peptide pools. A T‐cell response was considered positive when the frequency of IFN‐γ+ T cells was > 0.1% and at least 3‐fold above the background.

## 2 Results

An overview of clinical and virological findings is given in Table 1.

**TABLE 1:** Clinical and virological data.

General data				Virology											Clinical data								
Patient number	Sex	Age (years)	Malignancy	Phase of HBV infection	Anti‐HBc IgG	Anti‐HBs IgG > 1 IU/L	HBsAg qual.	HBsAg quant. (IU/mL)	HBeAg	Anti‐HBe IgG	HBV DNA	HBV DNA > 10 IU/mL	HBV genotype	Anti‐HEV IgG/IgM/RNA	Antiviral treatment	Alanine transaminase (U/L)^a^	Liver ultrasonography	ARFI median (m/s)	METAVIR fibrosis score	Ethanol consumption > 12 g/d	Body mass index (kg/m^2^)	Other diagnoses	Regular hepatologic follow‐up
01	m	38	WT	HBeAg‐neg CHB	+	−	+	20,222	−	+	+	3,010,000	D1	+/−/−	‐^b^	**82**	Mild steatotic p.d.	0.83	< 2	−	30.1		−
02	f	48	OS	HBeAg‐neg CHB	+	−	+	31,400	−	−	+	41,000,000	D1	−	‐^b^	**60**	Suspected fibrosis	1.74	**≥ 3**	−	18.0		+
03	f	46	GCT	HBeAg‐neg CHB	+	−	+	2,118	−	+	+	1,160,000	D1	−	‐^b^	**1,550**	Normal	1.37	< 2^c^	−	23.5		−
04	m	37	AML	HBeAg‐neg chron infection	+	−	+	4,053	−	+	−	−	n/a	+/−/−	ETV	29	Normal	1.27	< 2	−	24.2		+
05	m	52	ALL	HBeAg‐neg chron infection	+	4.71	+	18	−	+	+	251	D1	−	−	22	n/a	n/a	n/a	−	25.5		−
06	m	51	ALL	HBeAg‐neg chron infection	+	2.37	+	7	−	+	+	123	D1	−	−	28	Steatosis	1.31	< 2	40	26.1		−
07	f	41	ALL	HBeAg‐neg chron infection	+	−	+	937	−	−	+	< 20	n/a	−	TDF	**59**	Steatosis	1.19	< 2	−	30.1		+
08	f	47	ALL	HBeAg‐neg chron infection	+	−	+	4,533	−	+	+	849	D1	−	−	17	Normal	0.86	< 2	18	24.8		−
09	f	43	ALL	HBeAg‐neg chron infection	+	−	+	5,639	−	+	+	740	D1	−	−	17	Normal	0.92	< 2	−	23.7		−
10	m	39	ALL	HBeAg‐neg chron infection	+	−	+	554	−	−	−	−	n/a	−	ETV	**82**	Steatosis	1.41	≥ 2	−	32.4		+
11	m	39	ALL	HBeAg‐neg chron infection	+	−	+	13	−	+	+	103	D1	−	−	23	Normal	0.93	< 2	−	23.6		+
12	m	46	ALL	HBeAg‐neg chron infection	+	−	+	2,564	−	−	−	−	n/a	−	TDF	43	Normal	1.24	< 2	−	25.7		+
13	m	43	NHL	HBeAg‐neg chron infection	+	−	+	842	−	+	+	38	D1	−	−	29	Normal	1.05	< 2	−	27.2	AATD	+
14	f	37	ALL	HBeAg‐neg chron infection	+	−	+	40	−	+	−	−	n/a	−	‐^b^	10	Diffuse p.d.	1.60	≥ 3	80	22.9	d	−
15	f	39	NB	HBeAg‐neg chron infection	+	−	+	1,798	−	+	+	183	D1	−	−	31	Normal	1.02	< 2	−	18.2	CP	+
16	m	40	ALL	Resolved infection	+	83.72	−	n/a	−	−	n/a	n/a	n/a	−	−	42	Steatosis	0.88	< 2	−	25.5		−

*Note:* In the clinical data section, pathologic findings are in bold writing.

Abbreviations: AATD, α‐1‐antitrypsin deficiency (heterozygous); anti‐HBc, hepatitis B core antibody; anti‐HBe, hepatitis B e antibody; anti‐HBs, hepatitis B surface antibody; anti‐HEV, hepatitis E antibody; ALL, acute lymphoblastic leukaemia; AML, acute myeloid leukaemia; ARFI, acoustic radiation force impulse; CHB, chronic hepatitis B; CP, chronic pancreatitis; ETV, entecavir; GCT, granulosa cell tumour; HBeAg, hepatitis B e antigen; HBsAg, hepatitis B surface antigen; HBV, hepatitis B virus; METAVIR, Meta‐analysis of Histological Data in Viral Hepatitis; NB, neuroblastoma; NHL, non‐Hodgkin lymphoma; n/a, not applicable; OS, osteosarcoma; p.d., parenchymal damage; TDF, tenofovir disoproxil fumarate; WT, Wilms tumour; +, positive; −, negative/below detection limit (PCR < 10 IU/mL).

^a^ Alanine transaminase upper limit of normal: Female = 35 U/L; male = 50 U/L.

^b^ New treatment indication due to study findings.

^c^ ARFI median slightly above ≥ 1.34 attributed to very high ALT levels > 1,500 U/L not fibrosis; hence no signs of higher‐grade fibrosis.

^d^ Oesophageal varices Paquet II°, status post: Variceal haemorrhage, acute necrotising pancreatitis and multiorgan failure.

### 2.1 Clinical Follow‐Up

Current clinical practice guidelines from 2017 [24] recommend regular checks for ALT and HBV DNA levels, and liver ultrasonography. The assessment of clinical monitoring in our cohort showed that only six patients (38%) had been continuously monitored in recent years. Of the remaining 10 patients, four had at least received regular ALT and/or HBV DNA determinations, while six underwent no follow‐up at all. From a treatment status perspective, the four patients who were under antiviral treatment at the start of the study had an almost seamless follow‐up, with ultrasonography missing in only one case. In contrast, only three of the 12 untreated patients had a complete recent follow‐up.

**FIGURE 2:**
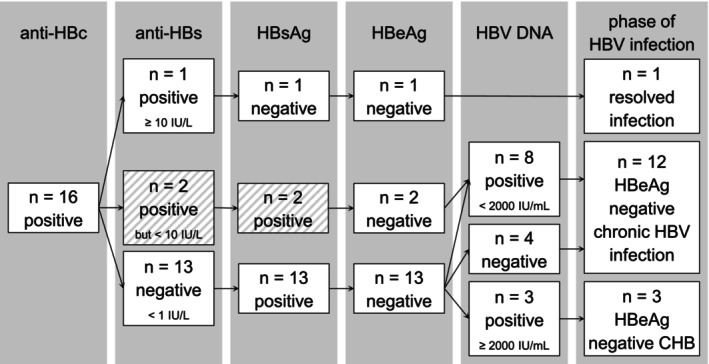
Virological diagnostics and phase of infection. The diagnostic algorithm and results are shown. The hatched boxes mark two patients (#05 and #06) with parallel detection of HBsAg and low anti‐HBs. They were equally classified as chronic HBV infection, since low anti‐HBs < 10 IU/L is not considered protective. Phases of HBV infection and the 2,000 IU/mL cut‐off value for serum HBV DNA were defined following the 2017 EASL clinical practice guidelines. Anti‐HBc, hepatitis B core antibody; anti‐HBs, hepatitis B surface antibody; CHB, chronic hepatitis B; HBeAg, hepatitis B e antigen; HBsAg, hepatitis B surface antigen; HBV, hepatitis B virus.

Most patients (*n* = 12; 75%) presented with HBeAg‐negative chronic HBV infection, including four patients who reached this inactive phase of HBV infection by receiving antiviral treatment; three (19%) showed an HBeAg‐negative CHB (chronic hepatitis B), and one (6%; patient #16) showed functional cure. An overview of participants' HBV infection status is shown in Figure 2.

Four patients (25%) of the cohort met the criteria for the initiation of antiviral therapy; among them were all three patients with HBeAg‐negative CHB (#01–03) plus one (#14) with previously known severe fibrosis and oesophageal varices due to severe ethanol abuse for 18 years.

Assessment of liver disease revealed relevant disease activity in all three patients with HBeAg‐negative CHB: One patient (#03) showed laboratory signs of an acute hepatitis flare with ALT elevation to 44‐fold the upper limit of normal, but no signs of structural damage to the liver on ultrasonography or elastography. The other two patients only had mild ALT elevation (below twice the upper limit of normal), but both showed structural changes in the liver, i.e., steatotic parenchymal damage on ultrasonography or severe fibrosis on elastography (median ≥ 1.55 m/s, METAVIR F ≥ 3), respectively. All three patients had very high levels of HBV DNA (> 10^6^ IU/mL), in contrast to low (*n* = 8; < 2,000 IU/mL) or below the detection limit (*n* = 4; < 10 IU/mL) levels in all other patients. In the remaining 13 patients (of whom ultrasonography and elastography could only be obtained in 12 patients), signs of marked liver disease activity or damage to the liver were present in only two patients (#10 and #14), and three (#06, #07, and #16) exhibited slight sonographic changes (steatosis). These findings were clinically assessed as either due to lifestyle factors rather than HBV infection or were previously known and stable: One patient (#14) qualified for the initiation of antiviral therapy due to ethanol toxic damage to the liver with signs of severe fibrosis on elastography (median ≥ 1.55 m/s, METAVIR F ≥ 3). Although she had no laboratory signs of chronic hepatitis (HBsAg weakly positive 40 IU/mL, HBV DNA undetectable, normal ALT) at the time of assessment, thereby formally not meeting the criteria for antiviral therapy according to the EASL guidelines, antiviral therapy was recommended. The reason for this clinical decision was that the patient had detectable HBV DNA (28 IU/mL) only 15 months earlier, thereby meeting the treatment criteria. The watch‐and‐wait approach with regular monitoring provided no realistic alternative considering the patient's poor compliance. The second patient with relevant liver disease (#10) showed steatosis and signs of significant fibrosis (median ≥ 1.34 m/s, METAVIR F ≥ 2) and slight ALT elevation below twice the upper limit of normal. In this case, fibrosis was diagnosed histologically more than 12 years ago in the context of an acute hepatitis flare and has shown no progression under antiviral therapy since then. The other three patients with mild steatosis on ultrasonography were overweight (#16, BMI ≥ 25.5 kg/m^2^), obese (#07, BMI ≥ 30 kg/m^2^), or consumed relevant amounts of alcohol (#06, 40 g/d).

### 2.2 Virologic Assessment

All patients tested positive for anti‐HBc. 13 patients were HBsAg‐positive and anti‐HBs negative. Patients #05 and #06, with low HBsAg levels (18 and 7 IU/mL, respectively), showed parallel detection of anti‐HBs at 4.7 and 2.4 IU/L. This level of < 10 IU/L is considered non‐protective and negative by most authors, but reveals a low level of active B‐cell response insufficient to saturate the circulating HBsAg. Patient #16 tested positive for anti‐HBs at 84 IU/L and negative for HBsAg and HBeAg, indicating a functional cure. HBV DNA levels ranged from negative to very high: Four patients had levels below the detection limit of < 10 IU/mL, three of whom were under antiviral treatment. One patient under antiviral treatment had a positive HBV PCR result but below the lower limit of quantification of < 20 IU/mL (#07), seven patients had low HBV DNA levels between 38 and 849 IU/mL (all treatment‐naive), and three patients presented with high HBV DNA levels between 1.16x10^6^ and 4.10x10^7^ IU/mL. HBV DNA was not tested in the patient who showed functional cure serologically. All 15 patients without a functional cure were HBeAg‐negative, 11 of whom also tested positive for anti‐HBe antibodies. Of the four patients without concomitant anti‐HBe positivity, three were under antiviral treatment, and the fourth was highly viraemic, fulfilling the criteria for the start of antiviral therapy. Regarding other hepatotropic viruses, all patients tested negative for HCV, HDV, and HEV, except for two patients who had IgG antibodies against HEV with negative IgM and RNA testing, suggesting a previous HEV infection.

**FIGURE 3:**
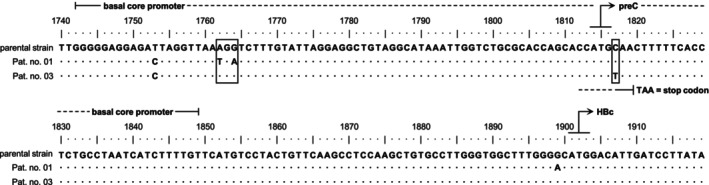
Mutations in the basal core promoter and preC (precore) region, respectively, found in two patient sera with high viral loads and HBeAg negativity. The boxes highlight mutations known to affect HBeAg expression. Nucleotide numbering follows the established way, starting at the EcoR1 restriction enzyme site. In the aligned patient strains, dots indicate the identical nucleotide and divergences are indicated by the respective letter. A complete genome sequence of the infecting strain, deposited in GenBank as Y07587 in 1996, served as the parental strain. HBc, hepatitis B core gene; preC, precore region.

Ten patients had sufficient HBV DNA levels (≥ 35 IU/mL) to determine the genotype of the infecting strain and were classified as sub‐genotype D1. These patients were all treatment‐naive. Primary drug resistance mutations in the reverse transcriptase domain of the P gene or immune escape mutations in the major hydrophilic region of the HBV surface protein (s99 to s169) [31] were not detected in any of the 10 patients. However, several non‐synonymous amino acid substitutions were identified, with none occurring in more than two patients. There was only one amino acid substitution (Y134F; patient #11) in the major hydrophilic region of the S protein. Sequencing and analysis of the BCP, preC, and Core regions were possible in two patients (#01 and #03) with high viral loads (> 10^6^ IU/mL), both of whom were HBeAg‐negative, anti‐HBe‐positive, and treatment‐naive. Both sequences showed a T1753C mutation in BCP. In #01, we found the A1762T/G1764A BCP mutation, and in #03, the C1817T mutation, which generates a stop codon in the preC sequence (Figure 3). The material was qualitatively insufficient for the third highly viraemic patient in the cohort (#02, HBeAg‐negative, without concomitant anti‐HBe). In five patients with low HBV DNA levels (123–849 IU/mL), none of the three alternative PCR methods produced amplification products for sequencing.

### 2.3 HBV‐Specific CD8+ T‐Cell Response

CD8+ T cells from all patients showed IFN‐γ secretion after stimulation with the CEF pool (positive control), indicating an overall intact functionality of the cells after transport and thawing. The median percentage of IFN‐γ+ CD8+ cells after incubation and stimulation with CEF peptides was 23.3%, with a rather wide scatter (minimum 0.9%; maximum 59.2%; IQR 31.4).

**FIGURE 4:**
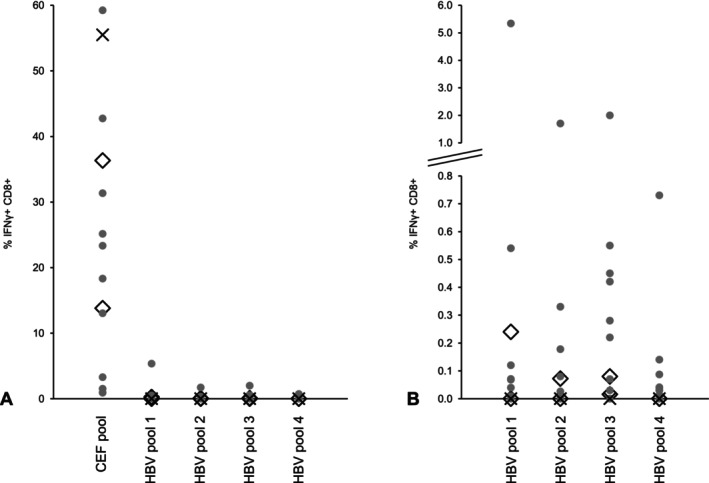
HBV‐specific CD8+ T‐cell response. Percentage of IFN‐γ+ CD8+ T cells after 14 days of antigen‐specific expansion in vitro. (A) Frequency of HBV‐specific T cells (HBV peptide pool 1–4) compared to frequency of CEF‐specific T cells. (B) Frequency of HBV‐specific T cells alone and upscaled. Individual patients are marked in groups corresponding to clinical/serological phases of their HBV infection. Symbol legend: ● = HBeAg‐negative chronic HBV infection; ◊ = HBeAg‐negative chronic hepatitis B; x = resolved/controlled infection. HBV, hepatitis B virus; CD, cluster of differentiation; CEF, human cytomegalovirus, Epstein–Barr virus, and influenza A virus; IFN‐γ, interferon γ.

The T‐cell responses to different HBV core peptide pools were as follows: After antigen‐specific expansion and stimulation, a T‐cell response (IFN‐γ+ CD8+ cells) to at least two of the four examined peptide pools was detectable in all but one (#16). This patient had a serologic profile of an anti‐HBs‐positive, resolved/controlled infection and showed no measurable HBV‐specific T‐cell reaction, while having 55.5% IFN‐γ+ CD8+ cells detectable after CEF‐specific expansion and stimulation. The frequency of HBV‐specific T cells against the four different peptide pools was low, with a percentage of IFN‐γ+ CD8+ cells below 0.8% in all but two patients (#08 and #13). Patients #08 and #13 both had untreated and stable HBeAg‐negative, low viraemic (849 and 38 IU/mL, respectively) chronic infections with no signs of clinical disease (liver enzyme elevation, abnormal sonographic findings, impaired hepatic function). Depending on the HBV peptide pool, they showed a percentage of HBV‐specific T cells up to > 5%. An overview of the T‐cell response to each peptide pool is shown in Figure 4. The detailed results grouped by clinical/serological phases of HBV infection are listed in Table 2. A formal statistical analysis of the differences between the patient groups was not possible owing to the small sample size.

**TABLE 2:** HBV‐specific T‐cell response and phase of HBV infection.

% CD8+ IFNγ+ T cells							Clinical data				
Patient number	CEF pool	HBV pool 1	HBV pool 2	HBV pool 3	HBV pool 4	Median HBV pool 1–4	Phase of HBV infection	HBsAg quant. (IU/mL)	HBV DNA (> 10 IU/mL)	Alanine transaminase (U/L)^a^	Antiviral therapy
01	36.318	0.000	0.072	0.016	0.000	0.008	HBeAg‐neg CHB	20,222	3,010,000	**82**	None^b^
03	13.810	0.240	0.000	0.080	0.000		HBeAg‐neg CHB	2,118	1,160,000	**1,550**	None^b^
04	23.335	0.000	0.080	0.070	0.000	0.055	HBeAg‐neg chron infection	4,053	—	29	ETV
06	59.239	0.120	0.000	0.550	0.040		HBeAg‐neg chron infection	7	123	28	None
07	18.330	0.040	0.178	0.220	0.000		HBeAg‐neg chron infection	937	< 20	**59**	TDF
08	25.160	5.340	1.700	0.450	0.140		HBeAg‐neg chron infection	4,533	849	17	None
09	1.510	0.000	0.000	0.280	0.087		HBeAg‐neg chron infection	5,639	740	17	None
10	13.030	0.071	0.000	0.000	0.030		HBeAg‐neg chron infection	554	—	**82**	ETV
11	31.350	0.540	0.330	0.420	0.730		HBeAg‐neg chron infection	13	103	23	None
12	3.270	0.010	0.026	0.030	0.041		HBeAg‐neg chron infection	2,564	—	43	TDF
13	42.750	0.068	0.000	2.000	0.014		HBeAg‐neg chron infection	842	38	29	None
14	0.903	0.000	0.000	0.070	0.031		HBeAg‐neg chron infection	40	—	10	None^b^
16	55.480	0.000	0.000	0.000	0.000	0.000	Resolved infection	n/a	n/a	42	None

*Note:* Median T‐cell responses aggregated after the phase of HBV infection.

Abbreviations: CEF, human cytomegalovirus, Epstein–Barr virus, and influenza A virus; CHB, chronic hepatitis B; ETV, entecavir; HBeAg, hepatitis B e antigen; HBsAg, hepatitis B surface antigen; HBV, hepatitis B virus; n/a, not applicable; TDF, tenofovir disoproxil fumarate; +, positive; ‐, negative/below detection limit (PCR < 10 IU/mL).

^a^ Alanine transaminase upper limit of normal: Female = 35 U/L; male = 50 U/L. Pathologic findings are in bold writing.

^b^ New treatment indication due to study findings.

## 3 Discussion

This study revealed that the majority of the patients experienced a relatively benign course of their chronic HBV infection, which had persisted for around 35 years. In 4/16 patients, this favourable course was supported by antiviral therapy with entecavir or tenofovir. However, regarding regular clinical monitoring of patients with chronic HBV infection, this study revealed large gaps, especially in patients not currently receiving antiviral treatment. This is important to note, as over 30 years after primary infection, close follow‐up has proven to remain necessary and justified for this cohort because of the known long‐term risks of chronic HBV infection, particularly liver fibrosis and hepatocellular carcinoma. In addition, the clinical follow‐up revealed highly relevant new findings in five patients, which have direct consequences on their lives: Three new indications for antiviral therapy of a highly replicative CHB and, on the positive side, HBsAg/anti‐HBs seroconversion in one patient, indicating a functional cure of the HBV infection. In one patient, monitoring revealed advanced alcoholic liver disease. As this patient was HBsAg‐positive and had detectable HBV DNA in the past, she was assigned to antiviral therapy according to the EASL guidelines, although she had no detectable HBV DNA in the serum at the time of our study.

The number of patients available for follow‐up has decreased since the last study of this unusual cohort approximately 25 years ago. Nevertheless, the newly gathered data confirm the previous conclusion that the course of HBV infection is most likely comparable to that of infections acquired during the perinatal period or early in life. This is remarkable because the children were up to 18 years old at the time of their primary infection. Loss of HBeAg has occurred in all patients throughout the 25 years since the initial follow‐up, when they were still HBeAg‐positive. In contrast, only one HBsAg/anti‐HBs seroconversion was observed. This is congruent with what is known about the natural history of chronic HBV infection acquired in early childhood [7, 32, 33]. The concept of “immune tolerance” of the adaptive immune system (i.e., T cells) towards viral antigens in early life and adolescence is increasingly being challenged. Instead, there is an alternative interpretation of a lacking capacity to mount a generally robust pro‐inflammatory environment in which the adaptive immune system can achieve viral control and clearance [34]. Considering this, it seems consistent to expect a similar clinical course of chronic HBV infection acquired under cytostatic treatment, as the general inflammatory responses are temporarily impaired under chemotherapy.

Sequencing of the BCP and preC regions in two cases with high viraemia (> 10^6^ IU/mL) revealed known mutations that can promote HBeAg negativity in highly viraemic patients by preventing preC translation by generating a stop codon (C1817T preC mutation) [35] or suppressing the transcription of preC (↓), while favouring the transcription of pregenomic (↑) mRNA. This results in reduced HBeAg expression, but enhanced virion production (A1762T/G1764A BCP double mutation) [36, 37, 38, 39]. These well‐known BCP mutations are often associated with another BCP mutation pattern, T1753V (V = C, A, or G; T1753C in our study, in both cases). All three BCP mutations have been reported to be associated with an increased risk of advanced liver disease during chronic HBV infection of genotype C [40] but also of genotype D [41]. Interestingly, these BCP mutations also affect the overlapping coding sequence of the multifunctional HBx (hepatitis B virus regulatory protein X) protein, causing amino acid exchanges in HBx that lead to changes in transactivation activity of HBx, which might contribute to carcinogenesis and development of hepatocellular carcinoma in genotypes C [42] and D [43].

Although HBeAg‐negative patients are believed to rarely have high viraemia, our study identified 3/16 such patients. The potential infectivity of these patients is of particular concern, because acute infection with such variants often causes severe or fulminant hepatitis [44]. Finding no known immune escape or drug resistance mutations in 10 patient sera hints at a certain level of long‐term persistent genomic stability over 30 years of chronic infection and supports the idea of “self‐normalising” mutational activity of HBV formulated by Tedder et al. [10] Regarding the analysis of drug resistance mutations, we could only examine treatment‐naive patients with no therapy‐induced selection pressure and our results are consistent with other studies that report low rates of drug resistance mutations in treatment‐naive patients [45].

The serologic finding of concurrent HBsAg and low‐titre anti‐HBs in two patients (#5 and #6) is puzzling. Parallel detection of HBsAg and anti‐HBs has been reported in a wide range of cases of chronic HBV infections, including (i) HBeAg‐negative patients with low viral load and appearance of (escape)‐mutations in the antigenic loop of HBsAg [46] and (ii) HBeAg‐positive patients with high viral load but without significant mutations within the antigenic loop of HBsAg [47]. Patients #5 and #6 do not fall under either constellation. Despite being HBeAg‐negative with low viral loads and low HBsAg levels, they do not exhibit any relevant mutations in the antigenic loop of HBsAg. However, it is possible that a minority of mutated HBsAg is being produced by integrated HBV DNA and is therefore not detectable in the wild‐type genomes of circulating HBV virions.

Investigation of the cytotoxic T‐cell response revealed a quantitatively weak HBV‐specific immunity, with a low frequency of CD8+ T cells reactive to HBV core peptides after in vitro expansion. This pattern is typically observed in chronic HBV infection, especially in HBeAg‐positive, highly viraemic chronic HBV infections [48, 49, 50]. Interestingly, all patients in our cohort in whom a weak T‐cell response could be examined were HBeAg‐negative, which is in line with previous reports [49, 51]. At the individual level, we had one patient with an acute hepatitis flare (relevant ALT elevation) at the time of sample collection, showing no higher CD8+ frequency than the other patients, which is consistent with earlier findings that the number of HBV‐specific T cells does not correlate with hepatic inflammatory activity [52].

## 4 Conclusion

More than 30 years after the primary infection, patients in this unique cohort exhibit a clinical course of chronic HBV infection that is most likely comparable to that of perinatally infected individuals. All of them have seroconverted from HBeAg‐positive chronic infection (formerly termed “immune tolerant” infection) to HBeAg‐negative chronic infection, with some of them entering the “immune active” phase of chronic infection with higher levels of HBV DNA and ALT elevation as a sign of increased hepatic inflammatory activity. Due to the small number of patients, the general validity of our virological findings and the assessment of T‐cell immunity is limited. However, re‐examining this special cohort after 30 years of chronic infection contributes to the limited knowledge on primary HBV infections under conditions of iatrogenic immunosuppression. Furthermore, our study underlines the necessity for regular clinical monitoring of chronically HBV‐infected patients, even decades after the primary infection, as there were a relevant number of patients requiring antiviral treatment due to study findings.

## Funding

The project was funded by the budgets of the participating institutions. This research was funded by the Deutsche Forschungsgemeinschaft (DFG, German Research Foundation) SFB 1021, 197785619, project B08 to D.G.; project TI 323/4‐1 to J.T.; the German Center for Infection Research (DZIF, partner site Giessen‐Marburg‐Langen), TTU Hepatitis, to D.G. The National Reference Centre for Hepatitis B Viruses and Hepatitis D Viruses at Justus Liebig University Giessen is supported by the German Ministry of Health through the Robert Koch Institute, Berlin.

## Conflicts of Interest

The authors declare no conflicts of interest.

## Data Availability

The data that support the findings of this study are available from the corresponding author upon reasonable request.
